# Discovery of a New Polymorph of 5-Methoxy-1*H*-Indole-2-Carboxylic Acid: Characterization by X-ray Diffraction, Infrared Spectroscopy, and DFT Calculations

**DOI:** 10.3390/molecules29102201

**Published:** 2024-05-08

**Authors:** Julia Polak, Julia Bąkowicz, Barbara Morzyk-Ociepa

**Affiliations:** 1 Institute of Chemistry, Faculty of Science and Technology, Jan Dlugosz University in Czestochowa, Armii Krajowej 13/15, 42-200 Czestochowa, Poland; juliapolak.05@gmail.com; 2 Faculty of Chemistry, Wroclaw University of Science and Technology, Wybrzeże Wyspiańskiego 27, 50-370 Wroclaw, Poland; julia.bakowicz@pwr.edu.pl

**Keywords:** 5-methoxyindole-2-carboxylic acid, polymorph, crystal structure, IR spectroscopy, DFT

## Abstract

This study presents a new 5-methoxy-1*H*-indole-2-carboxylic acid (MI2CA) polymorph investigated by single-crystal X-ray diffraction, infrared spectroscopy, and density functional theory (ωB97X-D) calculations employing two basis sets (6-31++G(d,p) and aug-cc-pVTZ). The compound crystallizes in the monoclinic system, space group P2_1_/c (a = 4.0305(2) Å, b = 13.0346(6) Å, c = 17.2042(9) Å, β = 91.871(5)°, Z = 4). In the crystalline structure, the formation of cyclic dimers via double hydrogen bonds O−H⋯O between MI2CA molecules was observed. Interactions between the NH groups of the indole rings and the adjacent methoxy groups, as well as C–H⋯O contacts, significantly influence the spatial arrangement of molecules. The results from DFT calculations, including dimeric and trimeric structures, agree well with the experimental structural and spectroscopic data. Analysis of the infrared spectra confirms the conclusions drawn from X-ray diffraction studies and reveals differences between the IR spectra of the newly obtained polymorph and that reported earlier in the literature. This comprehensive study sheds some light on the MI2CA polymorphism and is important for a potential pharmacological applications of this compound.

## 1. Introduction

Indole and its derivatives have a significant impact on physiological, biochemical, and metabolic processes in various organisms [1,2,3,4,5]. This study focuses on 5-methoxyindole-2-carboxylic acid (MI2CA), which is known for its potential neuroprotective properties in the context of stroke [6]. Convincing evidence highlights the promising effects of MI2CA on reducing ischemic area size, decreasing oxidative stress, and enhancing long-term potentiation (LTP) [7]. Additionally, MI2CA shows significant protective potential against human Aβ pathology in Alzheimer’s disease models [8] and is considered as a biomarker of malignant melanoma [9]. Increased concentrations of ester glucuronides and sulfates from compounds related to MI2CA have been observed in patients with advanced melanoma [10]. Moreover, MI2CA and its derivatives show promising potential in the treatment of diabetes [11].

Understanding intermolecular interactions in the crystals of these compounds is crucial due to their biological importance. For example, the crystal structure of indole-3-acetic acid (I3AA) shows the presence of O–H⋯O dimers [12,13], similar to those observed in indole-3-propionic acid (I3PA) [14]. Furthermore, the crystal structure of indole-3-carboxylic acid (I3CA) contains centrosymmetric cyclic dimers connected by O–H⋯O hydrogen bonds. Furthermore, the dimer units expand into a sheet structure through N–H⋯O interactions between the NH group of the pyrrole ring and the oxygen atom of the carboxylic group [15].

Our earlier studies on 5-methoxyindole-2-carboxylic acid (MI2CA) [16] and indole-2-carboxylic acid (I2CA) [17] revealed different roles of O–H and N–H groups, with the oxygen atom of the carboxylic group acting as an acceptor [16,17]. MI2CA crystallized in the monoclinic system, C2/c space group, with dimensions of a = 13.079(3) Å, b = 7.696(2) Å, c = 35.185 Å, β = 91.06(3)°, and Z = 16. In the asymmetric unit cell, there were two separate MI2CA molecules, which formed ribbons consisting of two independent molecular chains, connected by intermolecular hydrogen bonds O–H⋯O and N–H⋯O. No cyclic O−H⋯O dimers were present in the crystal [16]. In the present work, that structure has been denoted polymorph 1. In the case of I2CA, crystallographic analysis revealed that two chains of I2CA molecules formed a flat ribbon held together by intermolecular hydrogen bonds O–H⋯O and N–H⋯O [17]. Recent studies of three halogen derivatives, XI2CA, where X = F, Cl, and Br have shown the presence of cyclic dimeric structures with linear double O–H⋯O hydrogen bonds in the solid state [18].

I2CA and MI2CA form isostructural coordination polymers with Zn(II), Mn(II), and Cd(II) ions [19,20,21,22,23]. In these complexes, indole anions serve as bridging bidentate ligands. The coordination sphere around metal ions can be described as a distorted elongated octahedron in which four coordination sites are occupied by oxygen atoms of the carboxylate groups of four indole ligands (forming syn-skewed coordination structures), while the remaining two axial positions are occupied by two water molecules. As a result of interaction of I2CA, with iron a three-nuclear complex is formed [23]. Recent studies have focused on I2CA complexes with Ni(II) ions, in which the coordination environment around the metal is expanded with additional organic ligands [24]. Similar observations were previously reported for Cd(II) [25], Mn(II) [26], Zn(II) [27], and Ni(II) [28] ions, which further emphasize the diverse coordination behavior of these compounds.

Additionally, X-ray diffraction methods were used to study adducts obtained by combining I2CA with various organic compounds [29,30,31,32,33,34,35].

In this work, we present a new polymorph of MI2CA, denoted polymorph 2, with a special attention given to the structural aspects and intermolecular interactions in crystal. Infrared spectroscopy and DFT calculations provided detailed insights into the intermolecular hydrogen bonding patterns in MI2CA polymorph 2.

## 2. Results and Discussion

### 2.1. Crystal Structure of MI2CA Polymorph 2

**Figure 1:**
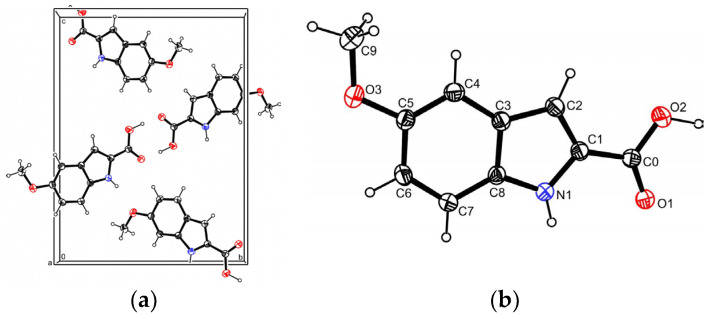
MI2CA polymorph 2: (**a**) four molecules in the unit cell; (**b**) molecular structure showing the atom numbering scheme. Displacement ellipsoids are drawn at the 25% probability level and H atoms are shown as small spheres of arbitrary radii.

The MI2CA polymorph 2 crystallizes in the monoclinic system, space group P2_1_/c, with the dimensions a = 4.0305(2) Å, b = 13.0346(6) Å, c = 17.2042(9) Å, and β = 91.871(5)°, with a Z-value of 4 (Figure 1a). Other crystallographic details are provided in Table S1 in the Supplementary Materials. The atom labeling in the compound’s formula is depicted in Figure 1b.

The conjugated benzene and pyrrole rings exhibit coplanarity, as evidenced by the torsional angles N1–C8–C3–C2 (−0.1(2)°) and N1–C8–C3–C4 (179.1(2)°), respectively. For comparison, in MI2CA polymorph 1, for two independent molecules A and B, these torsional angles were 0.4(1)°, 178.8(1)° for A, and −0.0(1)°, 179.3(1)° for B, respectively [16]. Additionally, in polymorph 2, the carboxyl group and the pyrrole ring are nearly coplanar, as shown by the torsional angles O1–C0–C1–N1 (−0.7(4)°) and O2–C0–C1–C2 (0.7(4)°). In polymorph 1, the corresponding torsional angles were 0.3(2)° and −0.2(2)° for A and 1.8(2)°, 0.2(2)° for B, respectively [16].

Table S2 in the Supplementary Materials presents selected experimental bond lengths and bond angles of MI2CA polymorph 2. According to the X-ray results, differences between the corresponding bond lengths in polymorph 2 and polymorph 1 [16] are mostly noted at the third decimal place. More significant variances are observed for bonds such as O2–C0, O3–C9, N1–C8, and N1–C1. In polymorph 2, these bonds are approximately 0.01 Å shorter than those in polymorph 1. Most of the corresponding bond angles in polymorphs 2 and 1 [16] closely resemble one another, differing by around one degree, except for three specific cases. In polymorph 2, angles C0–C1–C2 and O2–C0–C1 are approximately 3° larger than those in polymorph 1 [16]. Meanwhile, the angle O1–C0–C1 in polymorph 2 is about 3° smaller than in polymorph 1 [16]. These differences can be attributed to various intermolecular interactions present in both polymorphs.

**Figure 2:**
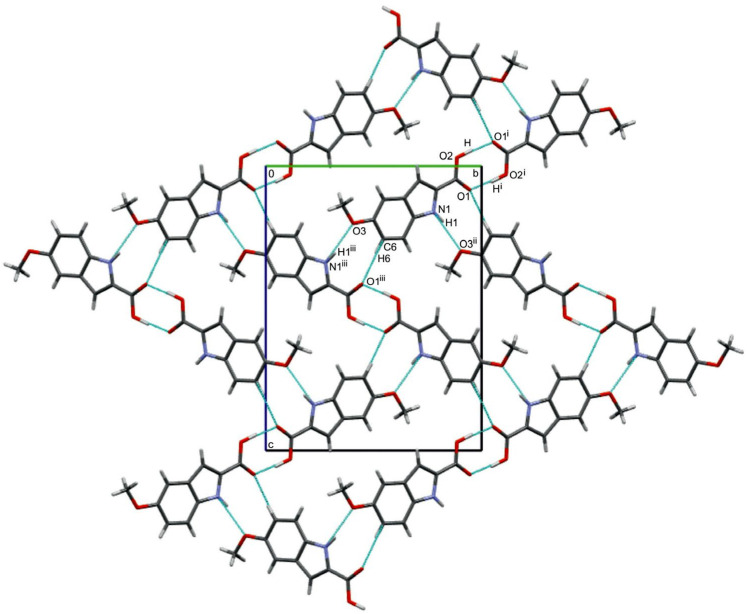
A fragment of the crystal lattice of MI2CA polymorph 2 with the O2−H⋯O1, N1−H1⋯O3, and C6−H6⋯O1 hydrogen-bonding arrangement viewed along the [100] axis. Corresponding symmetry codes are given in Table 1.

As demonstrated in the packing diagram (Figure 2), a pair of polymorph 2 molecules forms a centrosymmetric dimer connected by dual, nearly linear O–H⋯O hydrogen bonds, creating an eight-membered ring [R_2_^2^(8)]. The geometric parameters of the O2–H⋯O1^i^ hydrogen bond are presented in Table 1.

The O2–H⋯O1^i^ bond angle shows a slight deviation from linearity (by about 5(3)°). In I3AA and I3CA molecules, which also form dual O–H⋯O hydrogen bonds in the crystal. The corresponding O–H⋯O angles indicate smaller deviations from linearity, around 2° [13,15]. However, for I3PA, the corresponding O–H⋯O angles indicate larger deviations from linearity, about 9° [14]. In the crystal structure of polymorph 2, apart from dimers linked by strong O–H⋯O hydrogen bonds, there is a strong N1–H1⋯O3^ii^ hydrogen bond formed between the N1–H1 group of the pyrrole ring and the oxygen atom (O3) of the methoxy group from the adjacent dimeric unit, creating a nine-membered ring [R_2_^2^(9)]. In the crystal structure of polymorph 1, a strong N–H⋯O hydrogen bond was also present, but it was formed between the N–H group of the pyrrole ring and the oxygen atom of the carboxyl group [16]. In polymorph 2, in addition to the classical types of hydrogen bonds, a weak intermolecular C6–H6⋯O1^iii^ interaction was identified, with distances C6⋯O1 = 3.416(3) Å and H6⋯O1 = 2.57 Å, and the C6–H6⋯O1 angle of 151°, meeting the criteria specified for this type of hydrogen bond [36]. In this case also, a nine-membered ring [R_2_^2^(9)] is formed. In the case of polymorph 1, the C−H∙∙∙O bonds occur among different atoms, namely, C7A−H7A∙∙∙O2A, C7B−H7B∙∙∙O2A, and C9A−H9AA∙∙∙O3B, originating from two independent molecules of MI2CA. The geometric parameters of these hydrogen bonds are presented in Table S5 in the Supplementary Material.

**Figure 3:**
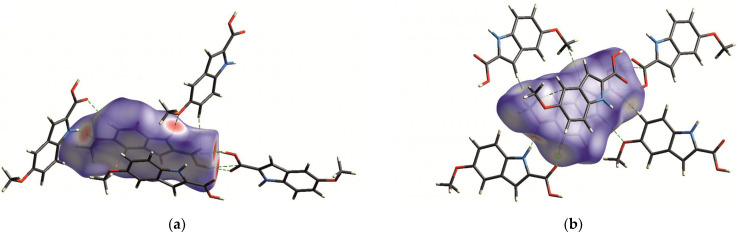
Hirshfeld surface mapped with d_norm_ for MI2CA polymorph 2: (**a**) highlighting the strong O2—H⋅⋅⋅O1 and N1—H⋅⋅⋅O3 hydrogen bonds; (**b**) emphasizing the weak C6—H6⋅⋅⋅O1 hydrogen bond. The red regions on the surface indicate distances shorter than the sum of van der Waals radii. White represents van der Waals separation, and blue indicates longer contacts.

Figure 3 shows the Hirshfeld surface mapped with d_norm_ [37] for MI2CA polymorph 2. This technique plays a pivotal role in identifying crucial intermolecular interactions within crystals and visualizing the interactions of a specific molecule with its neighboring molecules in a unified image.

The prominent large red spots, situated close to the hydrogen atoms of O2—H and N1—H groups, indicate the donors engaged in the prevailing O2—H⋅⋅⋅O1 and N1—H⋅⋅⋅O3 hydrogen bonds, as illustrated in Figure 3a. Additionally, conspicuous red spots adjacent to oxygen atoms O1 and O3 represent the acceptor atoms involved in these hydrogen bonds. Moreover, smaller red dots near the hydrogen H6 and oxygen O1 atoms in Figure 3b highlight the donor and acceptor of a weak C6—H6⋅⋅⋅O1 hydrogen bond (bearing in mind that spot size corresponds to interaction strength).

### 2.2. Molecular Structure and Intermolecular Interactions Present in the Crystal of MI2CA Polymorph 2, and Theoretical Results

**Figure 4:**
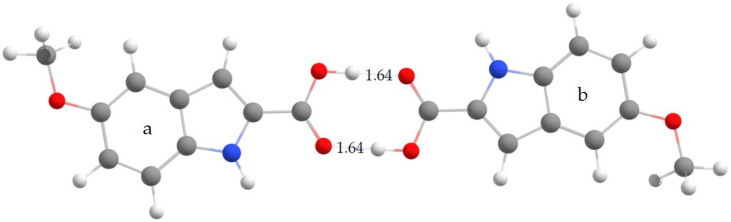
Computational model of dimer composed of the monomeric a and b units, showing intermolecular interactions and the H···O1 distances [in Å]. Calculations were performed using the ωB97X-D method with the 6-31++G(d,p) basis set. The atom numbering follows the scheme shown in Figure 1.

**Figure 5:**
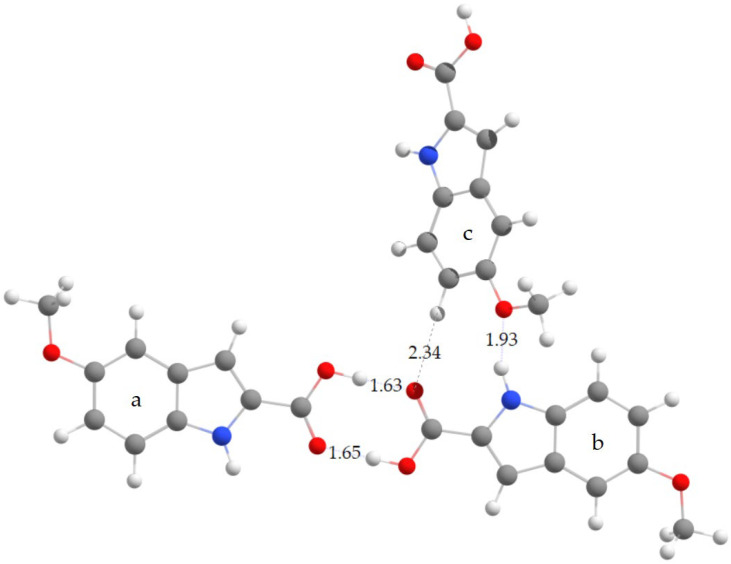
Computational model of trimer composed of the monomeric a, b, and c units, showing intermolecular interactions and distances, such as H···O1, H6···O1, and H···O3 [in Å]. These distances were computed using the ωB97X-D method with the 6-31++G(d,p) basis set. The atom numbering follows the scheme shown in Figure 1.

To investigate the molecular structure of 5MeBTA, geometry optimization was performed for both dimer (Figure 4) and trimer (Figure 5).

The dimer was created by combining two monomeric units, denoted a and b, taking into account the intermolecular O2—H⋅⋅⋅O1 hydrogen bonds. On the other hand, the trimer, composed of three monomeric units, a, b, and c, included all three intermolecular hydrogen bonds observed in the crystal: O2—H⋅⋅⋅O1, N1—H⋅⋅⋅O3, and C6—H6⋅⋅⋅O1.

Selected theoretical bond lengths and bond angles for dimer and trimer are listed in Table S2 in the Supplementary Materials. The optimized structure of dimer exhibits C_i_ symmetry (see Table 1), hence the a and b units have the same geometric parameters. The calculated structure of trimer has C_1_ symmetry. The corresponding structural parameters of the a, b, and c units are different.

**Figure 6:**
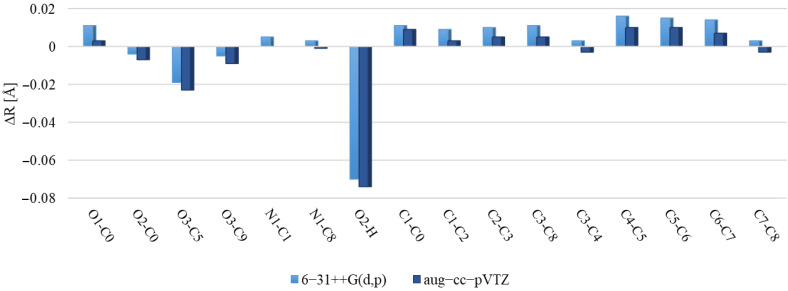
Differences between the ωB97X-D calculated bond lengths of dimer and the experimental values for MI2CA polymorph 2. Calculations were performed using two basis sets, 6-31++G(d,p) and aug-cc-pVTZ.

**Figure 7:**
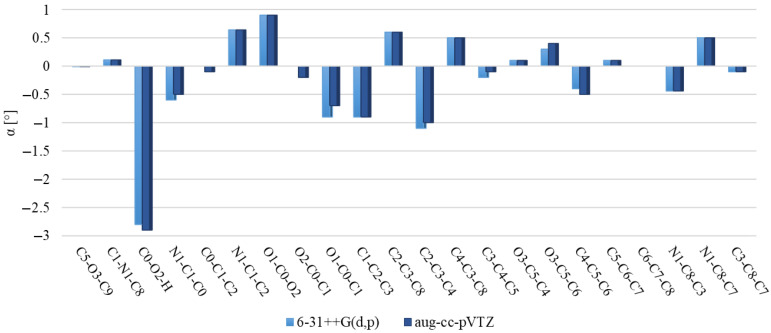
Differences between the ωB97X-D calculated bond angles of dimer and the experimental values for MI2CA polymorph 2. Calculations were performed using two basis sets, 6-31++G(d,p) and aug-cc-pVTZ.

**Figure 8:**
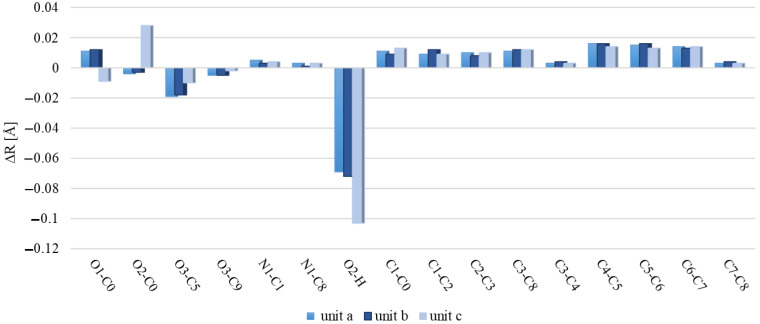
Differences between the calculated bond lengths of trimer and the corresponding experimental values for MI2CA polymorph 2. Calculations performed at the ωB97X-D/6-31++G(d,p) level of theory.

**Figure 9:**
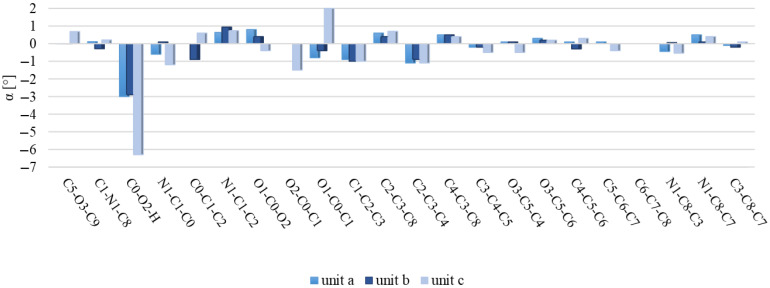
Differences between the calculated bond angles of trimer and the corresponding experimental values for MI2CA polymorph 2. Calculations performed at the ωB97X-D/6-31++G(d,p) level of theory.

Figure 6 and Figure 7 illustrate discrepancies between the calculated bond lengths and bond angles for dimer and those observed in the crystal structure of polymorph 2 of MI2CA. Figure 8 and Figure 9 show the differences between the respective experimental and theoretical bond lengths and bond angles for trimer.

As observed in Figure 6 and Figure 8, the results from calculations with the two model structures show similar trends for all bond lengths. The calculated C—C, C—N, and O1—C0 bond lengths are slightly overestimated, while the O2—C0, O3—C5, O3—C9, and O2—H bond lengths are underestimated with respect to the experiment. The aug-cc-pVTZ basis set provides better accuracy for the predicted C—C, C—N, and O1—C0 bond lengths, whereas the 6-31++G(d,p) basis set gives better agreement with experiment for O2—C0, O3—C5, O3—C9, and O2—H bond lengths. The noticeable differences between theoretical and experimental values are observed for the c unit of trimer, especially for the O1—C0, O2—C0, and O2—H bond lengths, as well as for the C0—O2—H bond angle. These discrepancies are attributed to the presence of a free carboxylic group in the c unit (Figure 5). As observed in Figure 7 and Figure 9, the differences between the calculated and experimental bond angles are approximately 1° (with the exception of the C0—O2—H bond angle, which is underestimated by about 3% in dimer and by 6° in the c unit of trimer).

For dimer, the average relative deviations (ARDs) between experimental and theoretical bond lengths (excluding C–H and N–H bonds) are 1.04% for the 6-31++G(d,p) and 0.88% for the aug-cc-pVTZ basis sets. The ARD values for bond angles (excluding C–C–H and C–N–H angles) are smaller, 0.44% in both basis sets. For trimer (excluding C–H and N–H bonds), the ARD values are: 1.04% in the a unit, 1.04% in the b unit, and 1.27% in the c unit. For bond angles (excluding C–C–H and C–N–H angles), ARDs are 0.42% in a, 0.39% in b, and 0.77% in the c unit. Additionally, we performed an analysis of correlations between experimental and theoretical geometrical parameters for dimer and trimer. The results are presented in Figures S1–S3 in the Supplementary Materials. Correlation coefficients (R^2^) approaching 1 indicate strong correlation, which means excellent agreement between theoretical predictions and experimental observations for molecular geometries.

All geometric parameters of intermolecular hydrogen-bonded bridges computed for dimer and trimer, as well as the experimental data for MI2CA polymorph 2, are collected in Table 1. As follows from this table, the two basis sets give very similar results for dimer. Calculations for trimer have revealed that the use of the ωB97XD functional with the 6-31++G(d,p) basis set almost replicates all experimental donor–acceptor distances for O2−H⋅⋅⋅O1, N1−H⋅⋅⋅O3, and C6−H6⋅⋅⋅O1 hydrogen bonds. The predicted bond angles are slightly larger than the experimental values, showing a deviation of less than 3% for the strong hydrogen bonds: O2−H⋅⋅⋅O1 and N1−H⋅⋅⋅O3. However, for the weak hydrogen bond C6−H6⋅⋅⋅O1, the bond angle shows a larger error, of about 8%, with respect to the experiment.

### 2.3. MIR Spectra of MI2CA Polymorph 2

**Figure 10:**
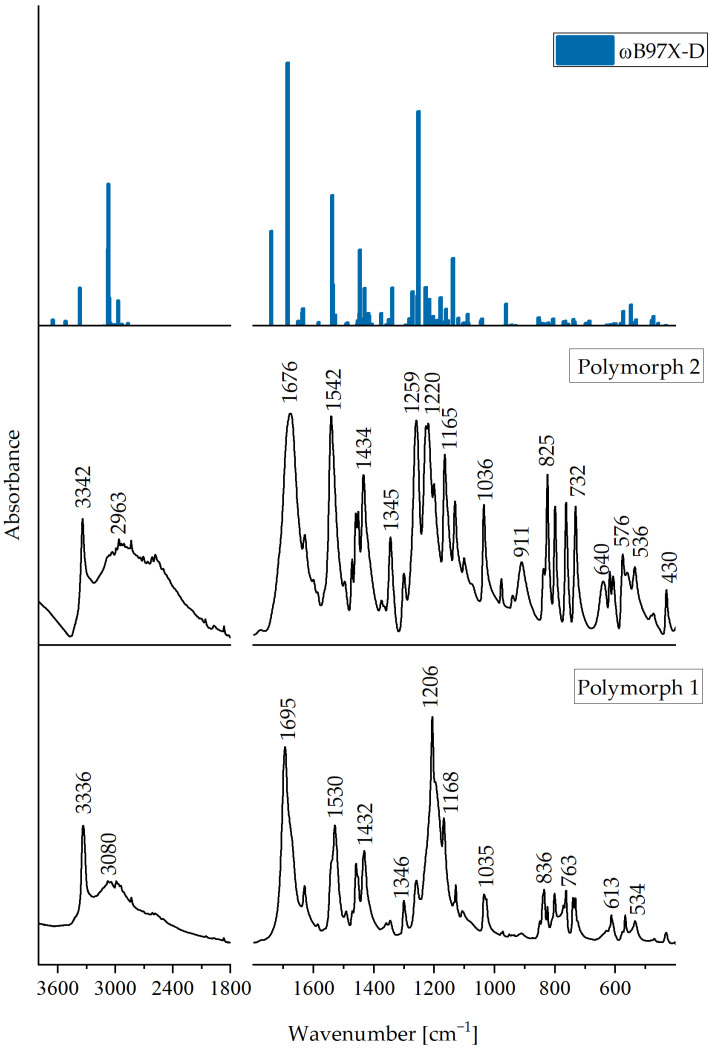
Comparison of the experimental IR spectra of MI2CA polymorph 1 [16] and polymorph 2 in the range 3800–400 cm^−1^ with the corresponding theoretical IR spectrum calculated for the trimer using the ωB97X-D method (blue line). The theoretical wavenumbers are scaled, as shown in Section 3.4.

The experimental infrared spectra of MI2CA polymorph 2 (this work) and MI2CA polymorph 1 [16] are presented in Figure 10. Additionally, the experimental spectrum of polymorph 2 is compared with the theoretical spectrum calculated for trimer using the ωB97X-D functional and the 6-31++G(d,p) basis set. The MI2CA trimer, consisting of 69 atoms (Figure 5), exhibits 201 vibrational modes (C_1_ point group), while the MI2CA dimer (Figure 4), containing 46 atoms, exhibits 132 vibrational modes (C_i_ symmetry). The computed frequencies for both the models were all real (no imaginary frequencies, which means that the calculated structures are stable). All theoretical wavenumbers and IR intensities for the MI2CA trimer as well as band assignments are summarized in Table S3 in the Supplementary Materials.

Table 2 lists the bands observed in the FT-IR spectrum of MI2CA polymorph 2, along with the corresponding wavenumbers calculated for trimer, which takes into account all intermolecular interactions occurring in the crystal. Band assignments have been made on the basis of the potential energy distribution (PED) calculated at the ωB97X-D/6-31++G(d,p) level of theory.

For the units a and b, each containing a free N–H group in trimer, the calculations predicted the ν(N–H) stretching vibrations at 3522 cm^−1^ and 3519 cm^−1^, respectively (Table S3 in the Supplementary Materials). It is expected that upon formation of an N–H⋯O hydrogen bond between units b and c (as depicted in Figure 5), the ν(N–H) stretching frequency should be lower than that for a free N–H group. Indeed, the ν(N–H) stretching vibration in the N–H⋯O hydrogen bond is predicted at 3373 cm^−1^. Therefore, in the experimental IR spectrum of polymorph 2, a sharp band at 3342 cm^−1^ has been assigned to the N–H stretching vibration. In the IR spectrum of polymorph 1, a similar band was observed at 3336 cm^−1^ [16,20]. These bands provide evidence for the presence of the N-H groups involved in intermolecular N–H⋯O hydrogen bonds in the two polymorphs. In both cases, the NH group acts as a hydrogen bond donor. However, it should be stressed that in polymorph 2, the hydrogen bond acceptor is the oxygen atom of the methoxy group (as indicated by this study), whereas in polymorph 1, it is the oxygen atom of the carboxylic group [16].

In the spectra of both polymorphs, a broad band is observed from about 3200 to 2000 cm^−1^. In this range, usually ν(O–H) stretching vibrations are observed. In the case of polymorph 2 with the cyclic O–H⋯O hydrogen bonds, this broad band has a greater relative intensity in comparison to polymorph 1, where O–H⋯O and N–H⋯O hydrogen bonds create a nine-membered ring [R_2_^2^(9)]. However, the definite assignment of the ν(O–H) stretching mode in the spectra is difficult due to the complicated character of these absorption bands. In this range of wavenumbers, the other bands resulting from the aromatic C–H stretching vibrations (3082, 3067, and 3033 cm^−1^) and the C–H stretching vibrations of the methyl group (2993–2835 cm^−1^) are also observed. Nevertheless, according to the calculations, the broad band with a maximum at 2963 cm^−1^ can be attributed to the stretching vibration of the O-H group participating in O–H⋯O hydrogen bonds, creating an eight-membered ring [R_2_^2^(8)].

The very strong band observed at 1676 cm^−1^ in the infrared spectrum of polymorph 2 closely matches the band predicted for the ν(C0=O1) stretching vibration at 1687 cm^−1^ (the calculated IR intensity of this band is very high, which is in agreement with the experiment). According to calculations, the ν(C0–O2) stretching vibration contributes mainly to the theoretical band at 1252 cm^−1^. In the spectrum of polymorph 2, a very strong band appears at 1259 cm^−1^, and can be assigned to this mode. Interestingly, in the spectra of halogen derivatives of I2CA, which form analogous cyclic dimers through O–H⋯O interactions, the ν(C=O) and ν(C–O) stretching vibrations were similarly assigned in the ranges 1659–1653 cm^−1^ and 1291–1254 cm^−1^, respectively [18]. On the other hand, for polymorph 1, which revealed a different pattern of intermolecular hydrogen bonds, the ν(C=O) and ν(C–O) stretching vibrations were observed at 1695 and 1206 cm^−1^, respectively [16,20].

The γ(O–H) out-of-plane bending vibrations in polymorph 2 are observed as a medium band at 911 cm^−1^, and this assignment is supported by the calculated wavenumber, 931 cm^−1^. This band is absent in the spectrum of polymorph 1.

In the IR spectrum of polymorph 2, a medium and broad band at 640 cm^−1^ is attributed to the γ(N–H)) out-of-plane bending vibration. This band is not visible in the spectrum of polymorph 1.

Various hydrogen bonding motifs lead to changes in the position and intensity of certain bands. For instance, in the spectrum of polymorph 2, a strong band is observed at 825 cm^−1^, whereas in polymorph 1, this band appears weaker. Additionally, polymorph 2 exhibits very strong bands at 1542 cm^−1^, 1227 cm^−1^, and 1220 cm^−1^, which are absent in the spectrum of polymorph 1. These discrepancies in the infrared spectra confirm the structural differences between the two polymorphs. Detailed assignments of all observed bands in the IR spectrum of polymorph 2 are compiled in Table 2.

## 3. Materials and Methods

### 3.1. Preparation of MI2CA Polymorph 2

The crystals of the MI2CA polymorph emerged unexpectedly during an attempt to synthesize a Co(III) complex of MI2CA. [Co(NH_3_)_6_]Cl_3_ (0.4 mmol; Pol-Aura, Olsztyn, Poland) was dissolved in 20 cm^3^ of distilled water and the mixture was stirred and heated for 15 min at a temperature of 45 °C. Then, 0.5 mmol of MI2CA (Sigma-Aldrich, Burlington, MA, USA) was dissolved in 20 cm^3^ of ethanol and added to the reaction mixture, which was heated to 45 °C and stirred for approximately 15 h. Afterwards, the mixture was left at room temperature in an open container. After approximately 7 days, polymorph 2 crystallized out.

Polymorph 1, as reported in [16], was obtained accidentally in the synthesis of the sodium salt of MI2CA. However, it should be emphasized that in this study, we also obtained the crystals of polymorph 1 by slow evaporation of a methanol solution of MI2CA, whereas its crystal structure was determined using X-ray crystallography (CCDC 2349632). This crystal turned out to be of better quality than the one in [16].

### 3.2. X-ray Structure Determination

X-ray diffraction data for single crystals of two polymorphs of MI2CA were obtained by means of a four-circle diffractometer equipped with a CCD detector using an ω-scan technique (Δω = 1°). The data collection and reduction, along with absorption corrections, were performed by the CrysAlis^Pro^ software package (version 1.171.42.49) [38]. The structure was solved by direct method using the SHELXS program and refined with the SHELXL2014 program [39,40]. All non-hydrogen atoms were located in a difference Fourier map and were treated anisotropically. The O-bound H atom was located in a ΔF map and refined without constraints. The hydrogen atoms of the methyl group at C9 were located in a difference Fourier map and refined as part of a rigid rotating group with a distance of 0.96 Å and *U*_iso_(H) = 1.5 *U*_eq_(C). The remaining H atoms, bonded to C and N atoms, were introduced at calculated positions as riding atoms, with C–H and N–H distances of 0.93 (C–H indole ring) and 0.86 Å, respectively, and U_iso_(H) values were constrained to be 1.2 U_eq_ (C, N). The crystal structures were visualized with the Ortep-3 and Mercury programs [41,42]. Motifs of hydrogen bonds have been described according to the recommendations in the literature [43].

Details of the data collection parameters, crystallographic data, and final agreement parameters of polymorph 2 and 1 (this work) are provided in Table S1 in the Supplementary Materials. Selected experimental geometrical parameters, including bond lengths [Å] and bond angles [°], with e.s.d. in parentheses, observed in MI2CA polymorph 1 (this work) are presented in Table S4. Experimental geometrical parameters of intermolecular interactions (distances [Å] and bond angles [°]) in MI2CA polymorph 1 are included in Table S5. CCDC 2277023 contains the supplementary crystallographic data for polymorph 2. CCDC 2349632 contains the crystallographic data for polymorph 1 obtained in this work.

### 3.3. Spectroscopic Measurements

The FT-infrared spectra of both MI2CA polymorphs (1 and 2) in the region 4000–400 cm^−1^ were measured using a Nicolet-Nexus (Thermo Electron Corporation, Waltham, MA, USA) spectrometer with KBr pellets.

### 3.4. Theoretical Methods

Computations were conducted using the Gaussian 16 program [44] within the framework of density functional theory (DFT) employing the ωB97X-D long-range corrected hybrid density functional with dispersion corrections [45]. The starting structural parameters for calculations were derived directly from X-ray diffraction (XRD) analysis of polymorph 2 of MI2CA, ensuring a reliable starting point for our computational investigations.

We employed the 6–31++G(d,p) [46,47] and aug-cc-pVTZ [48,49,50,51] basis sets because of their established good performance in accurate description of molecular geometries. The 6–31++G(d,p) basis set offers a balance between computational cost and accuracy of the results obtained, while the aug-cc-pVTZ basis set provides additional accuracy by incorporating more polarization and diffuse functions, which are important for capturing electron correlation effects. These basis sets offer a comprehensive description of the molecular structure of M2ICA polymorph 2. It is worth noting that while the geometry optimizations were performed with the two basis sets, the frequency calculations and PED analysis were carried out using the smaller basis set (6–31++G(d,p)) to alleviate computational burden.

Harmonic frequencies and infrared intensities were computed for the optimized structures. The ωB97X-D-calculated frequencies were scaled using three scale factors: 0.948 for frequencies ≥2000 cm^−1^, 0.953 for frequencies in the range from 2000 to 1000 cm^−1^, and 0.970 for frequencies <1000 cm^−1^, following recommendations [52].

To aid in the assignment of the infrared spectrum, potential energy distributions (PEDs) were determined using the FCART program (version 7.0) [53]. Additionally, normal modes were graphically visualized using the Chemcraft program [54].

## 4. Conclusions

Our research provides compelling evidence for the existence of a new polymorph 2 of MI2CA, which has been thoroughly characterized by single-crystal X-ray diffraction, infrared spectroscopy, and theoretical DFT calculations. The crystal structure of polymorph 2 revealed significant differences compared to the known polymorph 1 [16]. A distinguished feature of polymorph 2 is the formation of cyclic dimers connected by double hydrogen bonds O−H⋯O, which are not present in polymorph 1. In addition, the N−H⋯O intermolecular hydrogen bonds and C–H⋯O interactions are crucial in building the crystal structure of polymorph 2, as revealed by Hirshfeld analysis. The N–H⋯O hydrogen bonds were found in both polymorphs. In both cases, the NH group of the indole ring acts as a hydrogen bond donor. However, in polymorph 2, the hydrogen bond acceptor is the oxygen atom of the methoxy group, whereas in polymorph 1, it is the oxygen atom of the carboxylic group. In polymorphs 1 and 2, there are also C−H∙∙∙O interactions, but they occur between different atoms. In polymorph 2, there is the hydrogen bond C6−H6∙∙∙O1. In the case of polymorph 1, there are C7A−H7A∙∙∙O2A, C7B−H7B∙∙∙O2A and C9A−H9AA∙∙∙O3B hydrogen bonds that come from two independent MI2CA molecules.

A detailed experimental and theoretical analysis of the IR spectra of MI2CA polymorph 2 and comparison with the IR spectra of polymorph 1 revealed the characteristic differences, which confirm different patterns of intermolecular hydrogen bonds in these compounds. Thus, infrared spectroscopy demonstrates a straightforward method of distinguishing between the two polymorphic forms of MI2CA.

This study provides key information on the MI2CA polymorphism and reveals potential implications for future research on MI2CA pharmacological applications.

## Figures and Tables

**Table 1:** Experimental (X-ray) geometrical parameters of intermolecular interactions (distances [Å] and bond angles [°]) in MI2CA polymorph 2 and the corresponding theoretical parameters calculated for the dimer and trimer of MI2CA using the ωB97X-D method with the 6-31++G(d,p) (*) and aug-cc-pVTZ (**) basis sets.

	D−H	H···A	D···A	D−H···A
O2−H∙∙∙O1^i^, exp.	1.07(4)	1.56(4)	2.630(3)	175(3)
Dimer *, calc. C_i_	1.00	1.64	2.64	180
Dimer **, calc. C_i_	1.00	1.65	2.64	179
Trimer *, calc. C_1_	1.00	1.63/1.65	2.63/2.65	178.7/179.6
N1−H1∙∙∙O3^ii^, exp.	0.86	2.16	2.965(2)	156
Trimer *, calc. C_1_	1.02	1.93	2.91	160
C6−H6∙∙∙O1^iii^, exp.	0.93	2.57	3.416(3)	151
Trimer *, calc. C_1_	1.09	2.34	3.40	164

Symmetry codes: (i) 1 − x, 2 − y, −z; (ii) −x, 1/2 + y, 1/2 − z; (iii) −x, −1/2 + y, 1/2 − z.

**Table 2:** Experimental bands in the FT-IR spectrum of MI2CA polymorph 2 and the corresponding calculated wavenumbers ($\tilde{\nu }$ ^a^, cm^−1^) along with band assignments.

FT-IR	$\tilde{\nu }$ ^a^	Assignments ^b^
3342 s	3373	ν(N1H)
3082 m	3077	ν(C4H)
3067 m	3066	ν(C6H), ν(C7H)
3033 m	3054	ν(C7H), ν(C6H)
2993 m	3011	ν(Me)
2963 m	2973	ν(O2H)
2940 m	2947	ν(Me)
2835 m	2876	ν(Me)
1676 vs	1687	ν(C0=O1)
1629 m	1635	ν(R6)
1587 w	1583	ν(R6)
1542 vs	1537	ν(R5/R6), ν(C1C0)
1497 w	1491	ν(R5/R6), δ(N1H)
1472 w	1451	δ(Me)
1460 m	1446	δ(Me)
1452 m	1435	δ(Me)
1434 s	1431	ν(R5/R6)
1375 w	1419	δ(Me)
1345 m	1339	ν(R5), δ(O2H)
1300 w	1282	δ(R5/R6)
1259 vs	1252	ν(C0O2), ν(R5/R6)
1227 vs	1217	δ(C4H)
1220 vs	1205	ν(C5O3), δ(O3C5), δ(R5/R6)
1200 m	1191	δ(N1H), ν(R5/R6), ν(O3C5), δ(Me)
1165 s	1161	δ(Me), ν(O3C9)
1132 s	1128	δ(Me)
1101 w	1102	δ(C6H), δ(C7H)
1036 s	1043	ν(O3C9, ν(R6)
978 w	963	ν(R5), δ(R5)
940 w	945	δ(R6), ν(R6), ν(C5O3)
911 br	931	γ(OH)
838 m	855	γ(C6H), γ(C4H)
825 s	844	δ(R5/R6)
800 s	806	γ(C6H), γ(C7H)
763 s	766	γ(C2H)
732 s	738	δ(R5/R6), δ(COO)
640 br	698	γ(N1H)
618 m	618	δ(R5/R6), δ(COO)
607 m	613	τ(R5/R6)
576 m	599	τ(R5/R6)
561 m	573	δ(COO)
536 m	535	δ(C5O3C9)
473 w	458	δ(C5O3)
430 m	433	τ(R5/R6)

^a^ The calculated frequencies were scaled as shown in Section 3.4. ^b^ Assignments from PED calculated by FCART06 and verified by Chemcraft program. Abbreviations: br, broad; m, medium; s, strong; v, very; w, weak; ν, stretching; δ, in-plane bending; γ, out-of-plane bending; τ, torsion; Me, methyl group; 5R, five-membered ring; 6R, six-membered ring.

## Data Availability

The original contributions presented in the study are included in the article or Supplementary Materials. Further inquiries can be directed to the corresponding author.

## Supplementary Materials

The following supporting information can be downloaded at https://www.mdpi.com/article/10.3390/molecules29102201/s1. Table S1: Comparison of crystal data and structure refinement for polymorphs 2 and 1 (this work) of MI2CA; Table S2: Selected experimental (X-ray) geometrical parameters, including bond lengths [Å] and bond angles [°], with e.s.d. in parentheses, observed in polymorph 2, along with the corresponding theoretical parameters calculated for the dimer and trimer of MI2CA using the ωB97X-D method with the 6-31++G(d,p) (*) and aug-cc-pVTZ (**) basis sets; Figure S1: Correlations between the experimental and theoretical geometrical parameters calculated for dimer using the ωB97X-D method and two basis sets: 6-31++G(d,p) and aug-cc-pVTZ; Figure S2: Correlations between the experimental and theoretical bond lengths calculated for trimer using the ωB97X-D method and the 6-31++G(d,p) basis set; Figure S3: Correlations between the experimental and theoretical bond angles calculated for trimer using the ωB97X-D method and the 6-31++G(d,p) basis set; Table S3: Experimental (FT-IR) and theoretical wavenumbers ($\tilde{\nu }$ ^a^, cm^−1^) and infrared intensities (A^IR^, km·mol^−1^) calculated for trimer using the ωB97X-D method and the 6-31++G(d,p) basis set with band assignments; Table S4: Selected experimental geometrical parameters, including bond lengths [Å] and bond angles [°], with e.s.d. in parentheses, observed in MI2CA polymorph 1 (this work); Figure S4: Overall view and labeling of the atoms in two independent molecules (designated A and B) of polymorph 1 MI2CA. Displacement ellipsoids are drawn at the 50% probability level and H atoms are shown as small spheres of arbitrary radii; Table S5: Experimental geometrical parameters of intermolecular interactions (distances [Å] and bond angles [°]) in MI2CA polymorph 1. CCDC 2277023 contains the supplementary crystallographic data for new polymorph 2 of MI2CA, and CCDC 2349632 contains the supplementary crystallographic data for polymorph 1 of MI2CA (this work). These data can be obtained free of charge via https://www.ccdc.cam.ac.uk/structures/ or from the Cambridge Crystallographic Data center, 12 Union Road, Cambridge CB2 1EZ, UK: fax (+44)-1223-336-033 or e-mail deposit@ccdc.cam.ac.uk.
