# Supplementary material for: Discovery of a New Polymorph of 5-Methoxy-1H-Indole-2-Carboxylic Acid: Characterization by X-ray Diffraction, Infrared Spectroscopy, and DFT Calculations
Source: Molecules. 2024 May 8;29(10):2201. doi: 10.3390/molecules29102201 (PMC11123780; doi:10.3390/molecules29102201)
Supplement: Supplementary file 1 [file molecules-29-02201-s001.zip › molecules-2998628-supplementary.pdf]

molecules

SUPPLEMENTARY MATERIALS

Discovery of a New Polymorph of 5-Methoxy-1H-Indole-2-Carboxylic Acid: Characterization by X-Ray Diffraction, Infrared Spectroscopy, and DFT Calculations

Julia Polak 1, Julia Bąkowicz 2 and Barbara Morzyk-Ociepa 1,*

1 Institute of Chemistry, Faculty of Science and Technology, Jan Dlugosz University in Czestochowa,

Armii Krajowej 13/15, 42-200 Czestochowa, Poland; juliapolak.05@gmail.com

2 Faculty of Chemistry, Wroclaw University of Science and Technology, Wybrzeże Wyspiańskiego 27,

50-370 Wroclaw, Poland; julia.bakowicz@pwr.edu.pl

* Correspondence: bmorzyk-ociepa@o2.pl

CONTENTS

Table S1. Comparison of crystal data and structure refinement for polymorphs 2 and 1 (this work) of MI2CA.

Table S2. Selected experimental (X-ray) geometrical parameters, including bond lengths [Å] and bond angles [°], with e.s.d. in parentheses, observed in MI2CA polymorph 2, along with the corresponding theoretical parameters calculated for the dimer and trimer of MI2CA using the ωB97X-D method with the 6-31++G(d,p) (*) and aug-cc-pVTZ (**) basis sets.

Figure S1. Correlations between the experimental and theoretical geometrical parameters calculated for dimer using the ωB97X-D method and two basis sets: 6-31++G(d,p) and aug-cc-pVTZ.

Figure S2. Correlations between the experimental and theoretical bond lengths calculated for trimer using the ωB97X-D method and the 6-31++G(d,p) basis set.

Figure S3. Correlations between the experimental and theoretical bond angles calculated for trimer using the ωB97X-D method and the 6-31++G(d,p) basis set.

Table S3. Experimental (FT-IR) and theoretical wavenumbers ($\tilde{\nu}^a$, cm^{-1}), and infrared intensities (A^{IR}, $km \cdot mol^{-1}$) calculated for trimer using the ωB97X-D method and the 6-31++G(d,p) basis set with band assignments.

Table S4. Selected experimental geometrical parameters, including bond lengths [Å] and bond angles [°], with e.s.d. in parentheses, observed in MI2CA polymorph 1 (this work).

Figure S4. The overall view and labeling of the atoms in two independent molecules (designated as A and B) of polymorph 1 MI2CA. Displacement ellipsoids are drawn at the 50% probability level and H atoms are shown as small spheres of arbitrary radii.

Table S5. Experimental geometrical parameters of intermolecular interactions (distances [Å] and bond angles [°]) in MI2CA polymorph 1.

Table S1. Comparison of crystal data and structure refinement for polymorphs 2 and 1 (this work) of MI2CA.

	Polymorph 2	Polymorph 1 (this work)
Empirical formula	$C_{10}H_9NO_3$	$C_{10}H_9NO_3$
Formula weight	191.18	191.18
Crystal system	Monoclinic	Monoclinic
Space group	$P2_1/c$	C2/c
Unit cell dimensions (Å, °)	a = 4.0305(2)<br>b = 13.0346(6)<br>c = 17.2042(9)<br>β = 91.871(5)	a = 13.0260(4)<br>b = 7.6923(2)<br>c = 35.2181(11)<br>β = 90.984(3)
V (Å^3)	903.36(8)	3528.33(18)
Z (molecules/cell)	4	16
Density calculated (Mg m^{-3})	1.406	1.440
F(000)	400	1600
Crystal dimensions (mm)	0.06 x 0.06 x 0.44	0.50 x 0.50 x 0.05
Temperature (K)	298	298
Wavelength (Å)	0.71073 (Mo Kα)	0.71073 (Mo Kα)
θ range for data collection (°)	2.0 to 26.0	2.3 to 26.0
Reflections collected	2891	6163
Reflections independent	1759	3478
Reflections observed [I > 2σ(I)]	1139	2826
Number of refine parameters	132	263
R_{int}	0.023	0.014
R [I > 2σ(I)], wR_2 [all], S	0.051, 0.137, 1.06	0.044, 0.110, 1.08
$\Delta\rho_{max}$ and $\Delta\rho_{min}$ (eÅ^{-3})	0.18, -0.19	0.16, -0.18

Table S2. Selected experimental (X-ray) geometrical parameters, including bond lengths [Å] and bond angles [°], with e.s.d. in parentheses, observed in MI2CA polymorph 2, along with the corresponding theoretical parameters calculated for the dimer and trimer of MI2CA using the ωB97X-D method with the 6-31++G(d,p) (*) and aug-cc-pVTZ (**) basis sets.

Bond lengths, Bond angles	Exp.	Dimer*	Dimer**	Trimer*		
		a = b	a = b	a	b	c
O1-C0	1.225(3)	1.236	1.228	1.236	1.237	1.216
O2-C0	1.316(3)	1.312	1.309	1.312	1.313	1.344
O3-C5	1.382(3)	1.363	1.359	1.363	1.364	1.372
O3-C9	1.415(3)	1.410	1.406	1.410	1.410	1.413
N1-C1	1.371(3)	1.376	1.371	1.376	1.374	1.375
N1-C8	1.366(3)	1.369	1.365	1.369	1.367	1.369
O2-H	1.07(4)	1.000	0.996	1.001	0.998	0.967
C1-C0	1.448(3)	1.459	1.457	1.459	1.457	1.461
C1-C2	1.367(3)	1.376	1.370	1.376	1.379	1.376
C2-C3	1.416(3)	1.426	1.421	1.426	1.424	1.426
C3-C8	1.402(3)	1.413	1.407	1.413	1.414	1.414
C3-C4	1.410(3)	1.413	1.407	1.413	1.414	1.413
C4-C5	1.365(3)	1.381	1.375	1.381	1.381	1.379
C5-C6	1.405(3)	1.420	1.415	1.420	1.421	1.418
C6-C7	1.362(3)	1.376	1.369	1.376	1.375	1.376
C7-C8	1.401(3)	1.404	1.398	1.404	1.405	1.404
C5-O3-C9	117.81(19)	117.8	117.8	117.8	117.8	118.5
C1-N1-C8	108.69(19)	108.8	108.8	108.8	108.4	108.9
C0-O2-H	113.2(19)	110.4	110.3	110.2	110.3	106.9
N1-C1-C0	119.8(2)	119.2	119.3	119.2	119.9	118.6
C0-C1-C2	131.1(2)	131.1	131.0	131.1	130.2	131.7
N1-C1-C2	109.06(19)	109.7	109.7	109.7	110.0	109.8
O1-C0-O2	123.4(2)	124.3	124.3	124.2	123.8	123.0
O2-C0-C1	114.8(2)	114.8	114.6	114.8	114.8	113.3
O1-C0-C1	121.8(2)	120.9	121.1	121.0	121.4	123.8
C1-C2-C3	107.6(2)	106.7	106.7	106.7	106.6	106.6
C2-C3-C8	106.3(2)	106.9	106.9	106.9	106.7	107.0
C2-C3-C4	134.2(2)	133.1	133.2	133.1	133.3	133.1
C4-C3-C8	119.5(2)	120.0	120.0	120.0	120.0	119.9
C3-C4-C5	118.3(2)	118.1	118.2	118.1	118.1	117.8
O3-C5-C4	124.6(2)	124.7	124.7	124.7	124.7	124.1
O3-C5-C6	113.9(2)	114.2	114.3	114.2	114.1	114.1
C4-C5-C6	121.5(2)	121.1	121.0	121.6	121.2	121.8
C5-C6-C7	121.5(2)	121.6	121.6	121.6	121.5	121.1
C6-C7-C8	117.7(2)	117.7	117.7	117.7	117.7	117.7
N1-C8-C3	108.34(19)	107.9	107.9	107.9	108.4	107.8
N1-C8-C7	130.1(2)	130.6	130.6	130.6	130.2	130.5
C3-C8-C7	121.6(2)	121.5	121.5	121.5	121.4	121.7

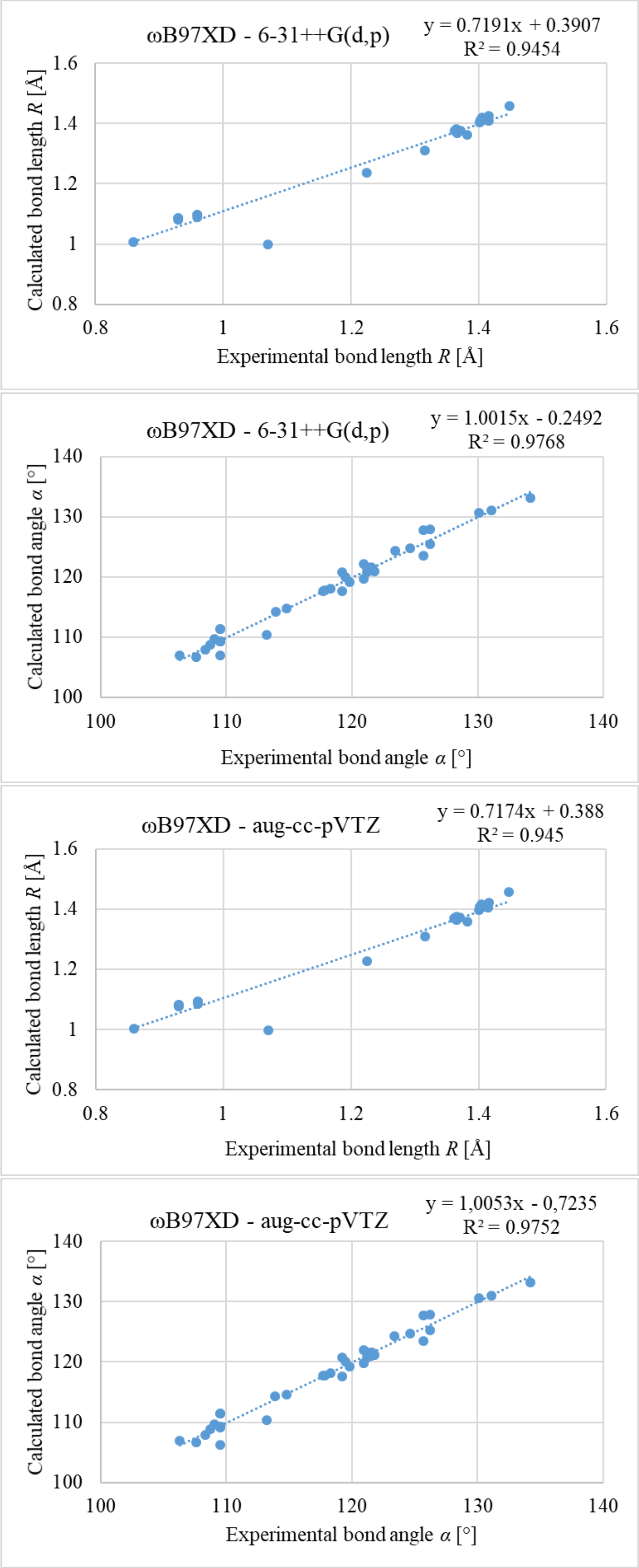

Figure S1. Correlations between the experimental and theoretical geometrical parameters calculated for dimer using the ωB97X-D method and two basis sets: 6-31++G(d,p) and aug-cc-pVTZ.

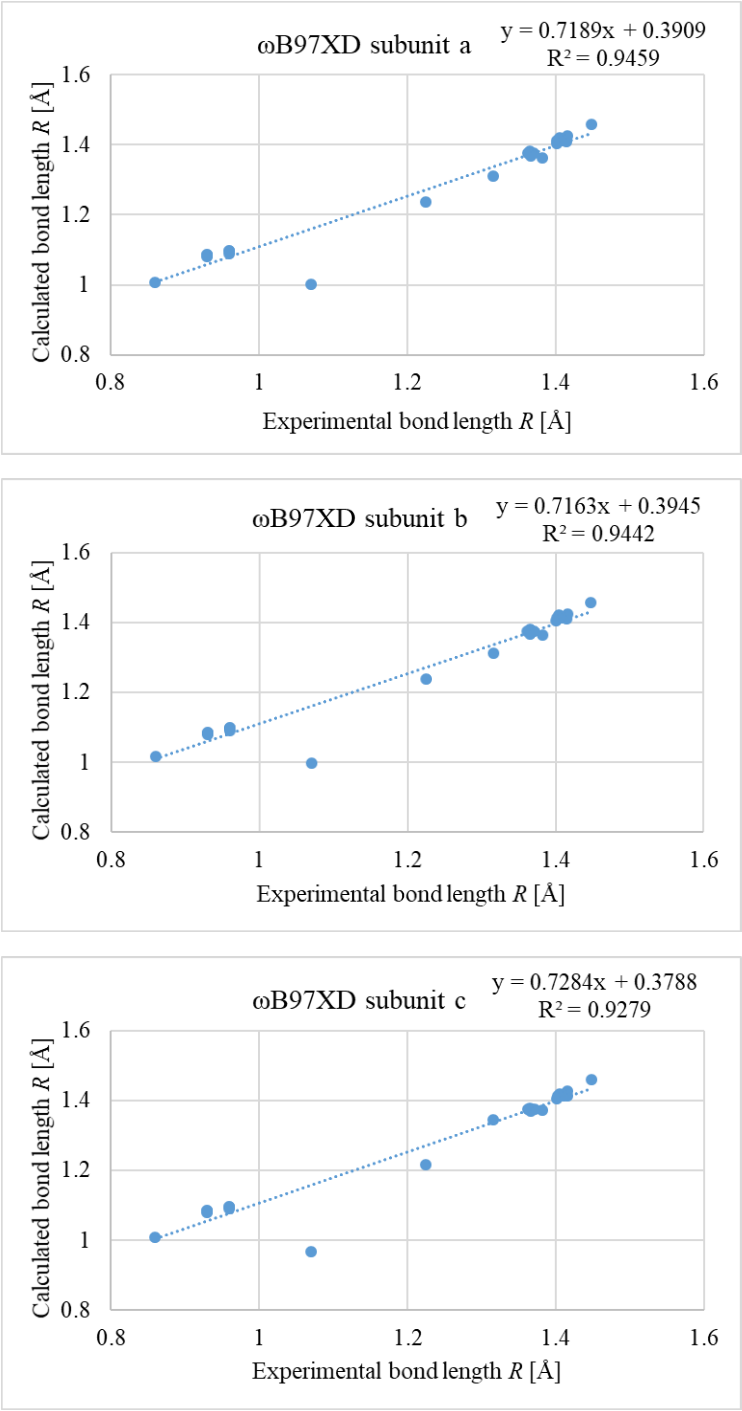

Figure S2. Correlations between the experimental and theoretical bond lengths calculated for trimer using the ωB97XD method and the 6-31++G(d,p) basis set.

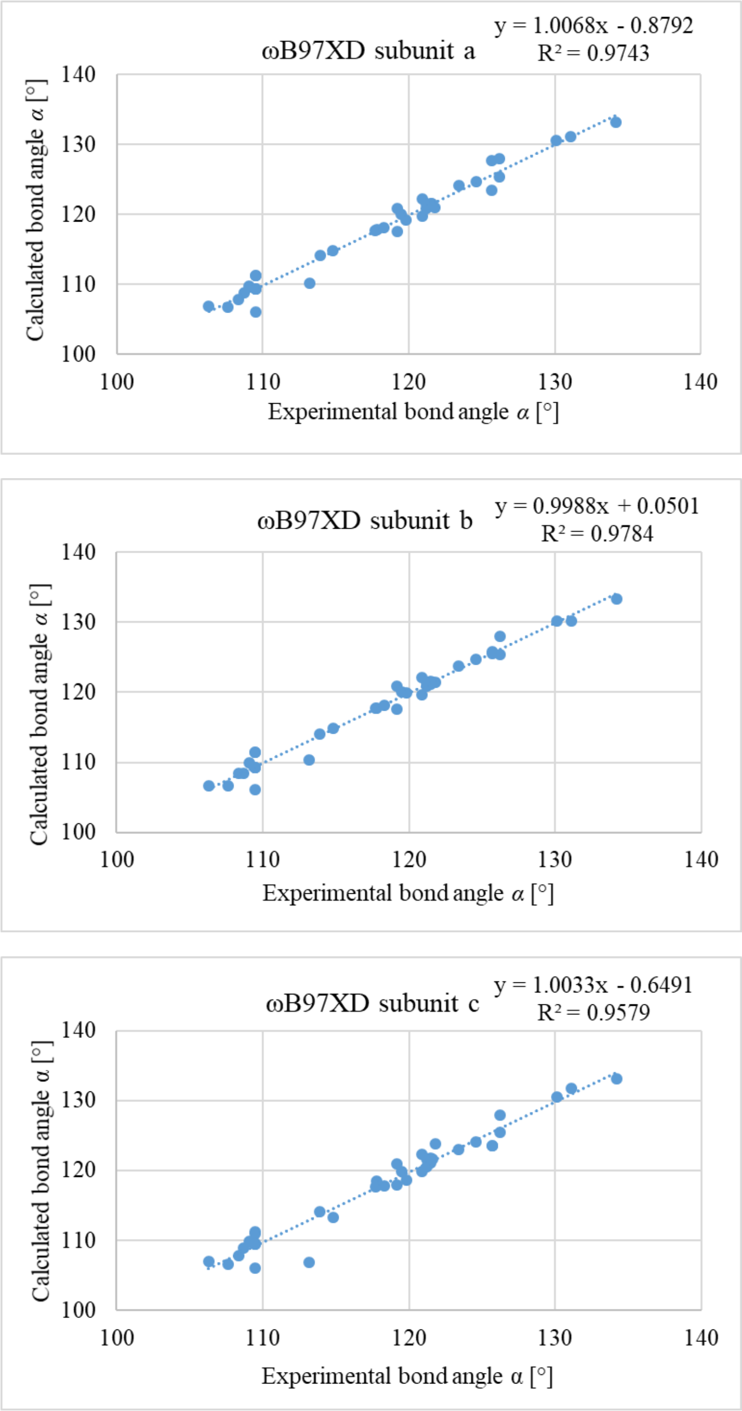

Figure S3. Correlations between the experimental and theoretical bond angles calculated for trimer using the ωB97XD method and the 6-31++G(d,p) basis set.

Table S3. Experimental (FT-IR) and theoretical wavenumbers ($\tilde{\nu}^a$, cm^{-1}), and infrared intensities (A^{IR}, $km \cdot mol^{-1}$) calculated for trimer using the ωB97X-D method and the 6-31++G(d,p) basis set with band assignments.

FT-IR	$\tilde{\nu}$ [a]	A^{IR}	Assignments [b]
	3655	153	ν(O2H)c
	3522	116	ν(N1H)a
	3519	118	ν(N1H)c
3342 s	3373	952	ν(N1H)b
	3124	0.17	ν(C2H)a
	3124	0.36	ν(C2H)c
	3123	0.01	ν(C2H)b
3082 m	3077	1930	ν(C4H)a,b
	3076	526	ν(C4H)b,a
	3076	16	ν(C4H)c
	3075	3552	ν(O2H)b,a
	3072	52	ν(C6H)a; ν(C7H)a
	3072	257	ν(C6H)b; ν(C7H)b
3067 m	3066	703	ν(C6H)c; ν(C7H)c
	3055	12	ν(C7H)b; ν(C6H)b
3033 m	3054	79	ν(C7H)c; ν(C6H)c
	3053	20	ν(C7H)a; ν(C6H)a
	3012	33	ν(Me)a
2993 m	3011	36	ν(Me)b
	3009	17	ν(Me)c
2963 m	2973	627	ν(O2H)a,b
2940 m	2947	30	ν(Me)c
	2935	40	ν(Me)a
	2934	40	ν(Me)b
2835 m	2876	43	ν(Me)c
	2869	54	ν(Me)a
	2869	69	ν(Me)b
	1739	595	ν(C0O1)c
1676 vs	1687	1650	ν(C0O1)a,b
	1652	34	ν(C0O1)a,b
	1635	89	ν(R6)c
1629 m	1635	109	ν(R6)a,b
	1633	3	ν(R6)a,b
	1585	6	ν(R6)c
1587 w	1583	25	ν(R6)a,b
	1582	0.24	ν(R6)a,b
1542 vs	1537	819	ν(R5/R6)a; ν(C1C0)a
	1536	259	ν(R5/R6)c; ν(C1C0)c
	1529	70	ν(R5)b; ν(C1C0)b
1497 w	1491	17	ν(R5/R6)b; δ(N1H)b
	1489	23	ν(R5/R6)a; ν(C1C0)a; δ(N1H)a
	1486	6	ν(R5/R6)c; δ(N1H)c; ν(C1C0)c
	1455	10	ν(R5)a,b; δ(O2H)a,b
	1452	36	δ(Me)c
1472 w	1451	39	δ(Me)b,c

FT-IR	$\tilde{\nu}$ [a]	A^{IR}	Assignments [b]
	1451	28	δ(Me)a; ν(R5/R6)a
	1447	84	δ(Me)b,c
1460 m	1446	477	δ(Me)a,b,c
	1443	17	δ(Me)a,b
1452 m	1435	45	δ(Me)c
	1434	10	δ(Me)a
	1434	9	δ(Me)b
1434 s	1431	238	ν(R5/R6)a,b
	1426	25	ν(R5/R6)c; δ(Me)c
	1422	24	δ(Me)b
1375 w	1419	80	δ(Me)a
	1416	66	δ(Me)c
	1413	5	δ(Me)b
	1408	15	ν(R5)a; δ(O2H)a, δ(N1H)a
	1376	80	ν(C0O2)c; ν(R5/R6)c; ν(C1C0)c
	1363	1	ν(R6)b
	1358	11	ν(R6)a
	1349	42	ν(R5/R6)c
	1344	0.26	ν(R5)a,b; δ(O2H)a,b
1345 m	1339	241	ν(R5)a,b; δ(O2H)a,b
	1295	8	ν(R5/R6)c; δ(O2H)c
1300 w	1282	50	δ(R5/R6)b
	1281	6	δ(R5/R6)a
	1272	216	δ(O2H)c; ν(R5/R6)c; ν(O3C9)c
	1256	193	ν(C0O2)b; ν(R5/R6)b; δ(C0O2)b
1259 vs	1252	1344	ν(C0O2)a; ν(R5/R6)a
	1227	243	ν(C5O3)b; δ(N1H)b; ν(O3C9)b; δ(C4H)b
	1218	155	δ(C4H)c
1227 vs	1217	169	δ(C4H)a
	1207	44	δ(C4H)b; δ(C7H)b
	1206	38	ν(R5/R6)a; δ(C7H)a
1220 vs	1205	63	ν(C5O3)c; δ(O3C9)c; δ(R5/R6)c
1200 m	1191	39	δ(N1H)b; ν(R5/R6)b; ν(O3C5)b; δ(Me)b
	1180	178	δ(N1H)c; ν(R5/R6)c; δ(Me)c
	1171	27	δ(N1H)a; ν(R5/R6)a; δ(Me)a
1165 s	1161	108	δ(Me)c; ν(O3C9)c
	1156	27	δ(Me)b; ν(O3C9)b
	1154	36	δ(Me)a; ν(R5/R6)a
	1138	424	δ(O2H)c; ν(C0O2)c; ν(R5)c
	1129	1	δ(Me)c
1132 s	1128	1	δ(Me)b
	1128	1	δ(Me)a
	1121	51	δ(C6H)c; δ(C7H)c
	1104	20	δ(C6H)a; δ(C7H)a
1101 w	1102	23	δ(C6H)b; δ(C7H)b
	1094	22	δ(C2H)b; δ(C4H)b
	1089	76	δ(C2H)a
	1087	16	δ(C2H)c

FT-IR	$\tilde{\nu}$ [a]	A^{IR}	Assignments [b]
	1044	32	ν(O3C9)b, ν(R6)b
	1044	32	ν(O3C9)a, ν(R6)a
1036 s	1043	46	ν(O3C9)c, ν(R6)c
	963	9	ν(R5)a; δ(R5)a; δ(C1C0)a
978 w	963	35	ν(R5)b; δ(R5)b
	962	138	γ(O2H)a,b
	955	2	ν(R5)c; δ(R5)c; ν(C1C0)c
	963	0.49	τ(R5/R6)c
	946	0.22	δ(R6)a,b; ν(R6)a,b
	946	6	δ(R6)a,b; ν(R6)a,b
940 w	945	8	δ(R6)c; ν(R6)c; ν(C5O3)c
	942	1	γ(C6H)b; γ(C7H)b
	942	0.0003	γ(C6H)a; γ(C7H)a
911 br	931	7	γ(O2H)a,b
838 m	855	55	γ(C6H)c; γ(C4H)c
	853	40	γ(C6H)b; γ(C4H)b
	852	38	γ(C6H)a; γ(C4H)a
825 s	844	20	δ(R5/R6)b
	838	10	δ(R5/R6)a
	836	17	δ(R5/R6)c
	822	22	γ(C6H)c; γ(C7H)c
	821	1	γ(C2H)a; γ(C4H)a
	820	3	γ(C2H)b; γ(C4H)b
	819	9	γ(C2H)c; γ(C4H)c
800 s	806	45	γ(C6H)b; γ(C7H)b
	806	17	γ(C6H)a; γ(C7H)a
	771	29	γ(C2H)b
	766	28	γ(C2H)a
763 s	766	32	γ(C2H)c
	764	4	δ(COO)a,b; ν(C5O3)a,b
	760	1	ν(C5O3)a,b; δ(R5/R6)a,b
	756	14	δ(R5/R6)c; ν(C5O3)c
732 s	738	43	δ(R5/R6)a,b; δ(COO)a,b
	735	19	τ(R5/R6)b,c; γ(C2H)c
	735	28	τ(R5/R6)c; γ(C2H)c
	733	10	τ(R5/R6)a; γ(C0C1)a; γ(C5O3)a
	722	1	δ(COO)a,b
640 m, br	698	19	γ(N1H)b
	686	33	δ(COO)c
	630	9	τ(R5/R6)c; γ(CCO)c
618 m	618	11	δ(R5/R6)b; δ(CCO)b
	615	2	τ(R5/R6)b
	613	1	τ(R5/R6)c; γ(O2H)c
607 m	613	0.34	τ(R5/R6)a
	612	1	τ(R5/R6)c; γ(C5O3)c; γ(COO)c
	604	16	δ(R5/R6)c; δ(C5O3)c; δ(COO)c
576 m	599	18	τ(R5/R6)a
	582	19	τ(R5/R6)b

FT-IR	$\tilde{\nu}$ [a]	A^{IR}	Assignments [b]
561 m	573	95	δ(COO)a,b
	562	1	δ(COO)a,b
	552	4	δ(COO)c
	548	134	γ(O2H)c
536 m	535	25	δ(C5O3C9)c;
	533	6	δ(C5O3C9)a,b
	531	40	δ(C5O3C9)a,b
	479	38	γ(N1H)c
	474	3	δ(C5O3)a,b
	472	61	γ(N1H)a
	468	0.06	δ(C5O3)a,b
473 w	458	20	δ(C5O3)c
430 m	433	5	τ(R5/R6)b
	430	2	τ(R5/R6)c
	429	1	τ(R5/R6)a
	393	9	δ(C5O3C9)b; δ(R5/R6)b; ν(H···O1)a,b
	388	43	δ(C5O3C9)a; δ(R5/R6)a; ν(H···O1)a,b
	379	0.21	τ(R5/R6)b,c; γ(C5O3)b,c; γ(O2H)c
	378	1	τ(R5/R6)b,c; γ(C5O3)b,c; γ(H···O3)b,c
	377	0.01	τ(R5/R6)a; γ(C5O3)a; γ(H···O3)b,c
	370	1	δ(C5O3C9)c; δ(R5/R6)c, δ(CCO)c
	345	0.21	δ(CCO)a,b; δ(R5/R6)a,b
	327	24	δ(COO)a,b
	320	0.36	δ(CCO)c; δ(COO)c
	281	0.24	γ(C5O3)c
	271	0.07	τ(R5/R6)a,b; γ(C5O3)a,b
	270	1	τ(R5/R6)a,b; γ(C5O3)a,b
	256	1	τ(R5/R6)c; τ(C5O3)c; γ(Me)c
	249	71	τ(R5/R6)b; δ(C5O3C9)b; γ(C1C0)c
	247	15	τ(R5/R6)b; δ(C5O3C9)b
	246	6	δ(C5O3C9)a
	239	7	δ(H···O1)a,b
	220	8	δ(C5O3C9)c
	189	0.26	τ(R5/R6)b; γ(C5O3)b
	184	1	τ(R5/R6)a; γ(C5O3)a
	183	1	δ(H···O1)a,b
	181	2	τ(R5/R6)c
	168	9	δ(H···O1)a,b
	123	2	δ(H···O3)b,c
	113	0.12	γ(C5O3)a,b; τ(R6/R5)a,b
	103	7	γ(C5O3)c; ν(H···O3)b,c; τ(R5/R6)b,c
	101	3	γ(C5O3)c; δ(H···O3)b,c; τ(R5/R6)b,c; γ(H···O3)b,c
	96	0.16	τ(R6/R5)a,b
	93	2	τ(R6/R5)a,b
	91	1	ν(H···O1)a,b; τ(R6/R5)a,b
	88	1	τ(R5/R6)c
	81	0.36	ν(H···O3)b,c; δ(H···O3)b,c
	76	0.18	τ(R5/R6)a,b

FT-IR	$\tilde{\nu}$ [a]	A^{IR}	Assignments [b]
	71	0.31	γ(C5O3)a; τ(R5/R6)a
	68	1	τ(R6/R5)c; δ(H···O3)b,c; γ(C5O3)b
	52	0.3	ν(H···O3)b,c; γ(COO)b
	43	1	δ(H···O3)b,c; γ(COO)b
	38	0.25	δ(H···O3)b,c; δ(R5)b
	35	0.12	τ(R5)a,b; γ(C1C0)a; δ(H···O3)b,c
	25	0.25	δ(H···O3)b,c, γ(COO)b
	22	0.17	δ(H···O3)b,c; γ(CCO)a,b, δ(H···O1)a,b
	17	0.05	δ(H···O3)b,c; γ(C5O3)c
	9	0.02	γ(COO)b; δ(H···O3)b,c
	7	0.23	γ(COO)b; δ(H···O3)b,c
	2	0.01	τ(R5)b

Abbreviations: a, b, c, units in the trimer; br, broad; m, medium; s, strong; v, very; w, weak; ν, stretching; δ, in-plane bending; γ, out-of-plane bending; τ, torsion; Me, methyl group; 5R, five-membered ring; 6R, six-membered ring. [a] The calculated frequencies were scaled, as shown in Section 3.4. [b] Assignment from PED calculated by FCART06 and verified by Chemcraft program.

Table S4. Selected experimental geometrical parameters, including bond lengths [Å] and bond angles [°], with e.s.d. in parentheses, observed in MI2CA polymorph 1 (this work).

O1A-C0A	1.224(2)	O1B-C0B	1.221(2)
O2A-C0A	1.319(2)	O2B-C0B	1.32(2)
O3A-C5A	1.375(2)	O3B-C5B	1.373(2)
O3A-C9A	1.419(2)	O3B-C9B	1.418(2)
N1A-C1A	1.379(2)	N1B-C1B	1.375(2)
N1A-C8A	1.372(2)	N1B-C8B	1.376(2)
O2A-HO2A	0.92(2)	O2B-HO2B	0.91(2)
C0A-C1A	1.456(2)	C0B-C1B	1.457(2)
C1A-C2A	1.369(2)	C1B-C2B	1.37(2)
C2A-C3A	1.414(2)	C2B-C3B	1.418(2)
C3A-C8A	1.408(2)	C3B-C8B	1.404(2)
C3A-C4A	1.415(2)	C3B-C4B	1.415(2)
C4A-C5A	1.368(2)	C4B-C5B	1.365(2)
C5A-C6A	1.414(2)	C5B-C6B	1.406(3)
C6A-C7A	1.366(2)	C6B-C7B	1.367(3)
C7A-C8A	1.397(2)	C7B-C8B	1.398(3)
C5A-O3A-C9A	117.2(1)	C5B-O3B-C9B	117.8(1)
C1A-N1A-C8A	108.0(1)	C1B-N1B-C8B	108.2(1)
HO2A-O2A-C0A	110(2)	HO2B-O2B-C0B	114(2)
N1A-C1A-C0A	122.2(1)	N1B-C1B-C0B	122.0(1)
C0A-C1A-C2A	128.2(2)	C0B-C1B-C2B	128.7(2)
N1A-C1A-C2A	109.6(1)	N1B-C1B-C2B	109.2(1)
O1A-C0A-O2A	122.8(2)	O1B-C0B-O2B	122.8(2)
O2A-C0A-C1A	111.8(1)	O2B-C0B-C1B	111.9(1)
O1A-C0A-C1A	125.5(2)	O1B-C0B-C1B	125.3(2)
C1A-C2A-C3A	107.5(1)	C1B-C2B-C3B	107.8(1)
C2A-C3A-C8A	106.5(1)	C2B-C3B-C8B	106.2(1)
C2A-C3A-C4A	133.5(2)	C2B-C3B-C4B	133.8(2)
C4A-C3A-C8A	120.0(1)	C4B-C3B-C8B	120.0(1)
C3A-C4A-C5A	118.0(1)	C3B-C4B-C5B	118.1(2)
O3A-C5A-C4A	124.6(1)	O3B-C5B-C4B	125.0(2)
O3A-C5A-C6A	114.3(1)	O3B-C5B-C6B	113.8(2)
C4A-C5A-C6A	121.1(2)	C4B-C5B-C6B	121.2(2)
C5A-C6A-C7A	121.9(2)	C5B-C6B-C7B	121.9(2)
C6A-C7A-C8A	117.7(2)	C6B-C7B-C8B	117.6(2)
N1A-C8A-C3A	108.5(1)	N1B-C8B-C3B	108.5(1)
N1A-C8A-C7A	130.3(2)	N1B-C8B-C7B	130.3(2)
C3A-C8A-C7A	121.3(1)	C3B-C8B-C7B	121.2(2)

The atom numbering follows the scheme shown in Figure S4.

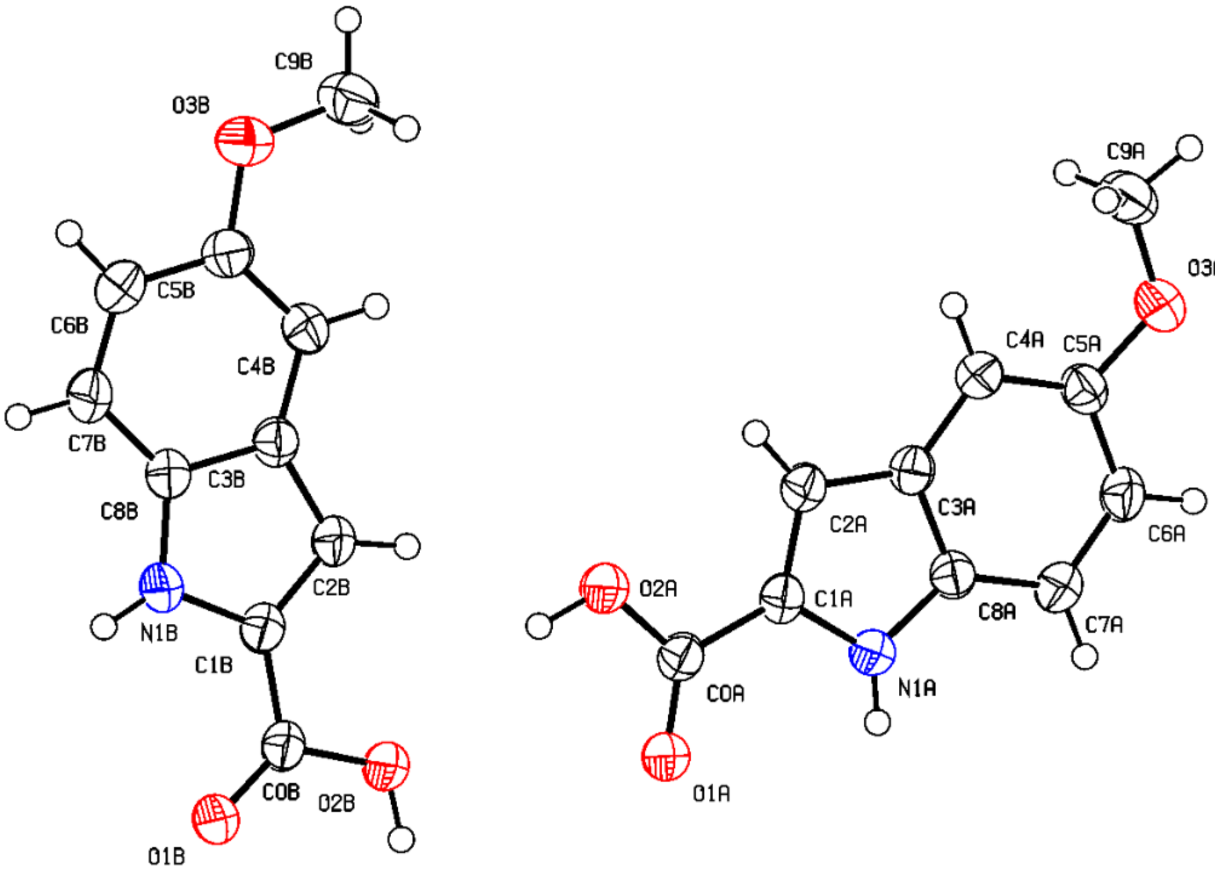

Figure S4. The overall view and labeling of the atoms in two independent molecules (designated as A and B) of polymorph 1 MI2CA. Displacement ellipsoids are drawn at the 50% probability level and H atoms are shown as small spheres of arbitrary radii.

Table S5. Experimental geometrical parameters of intermolecular interactions (distances [Å] and bond angles [°]) in MI2CA polymorph 1.

	**D–H**	**H···A**	**D···A**	**D–H···A**
O2A–Ho2A···O1B^{i}	0.92(2)	1.85(2)	2.7025(18)	154(2)
N1A–H1A···O1B^{ii}	0.86	2.21	3.0522(18)	165
N1B–H1B···O1A^{i}	0.86	2.22	3.0675(18)	169
O2B–Ho2B···O1A^{ii}	0.91(2)	1.78(3)	2.6504(18)	158(2)
C9A–H9AA···O3B^{iii}	0.96	2.53	3.382(3)	149
C7A–H7A···O2A^{iv}	0.93	2.51	3.365(2)	153
C7B–H7B···O2B^{v}	0.93	2.41	3.324(2)	168

Symmetry codes: (i) 3/2-x,1/2-y,1-z; (ii) 1-x,-y,1-z; (iii) 3/2-x,-1/2+y,3/2-z; (iv) -1/2+x,-1/2+y,z; (v) 1/2+x,1/2+y,z.
